# Proteinortho: Detection of (Co-)orthologs in large-scale analysis

**DOI:** 10.1186/1471-2105-12-124

**Published:** 2011-04-28

**Authors:** Marcus Lechner, Sven Findeiß, Lydia Steiner, Manja Marz, Peter F Stadler, Sonja J Prohaska

**Affiliations:** 1 RNA Bioinformatics Group, Department of Pharmaceutical Chemistry, Philipps-University Marburg, Marbacher Weg 6, D-35037 Marburg, Germany; 2 Bioinformatics Group, Department of Computer Science, Härtelstraße 16-18, D-04107, Leipzig, Germany; 3 Bioinformatics in EvoDevo Group, Department of Computer Science, University of Leipzig, Härtelstraße 16-18, D-04107, Leipzig, Germany; 4 Interdisciplinary Center for Bioinformatics, University of Leipzig, Härtelstraße 16-18, D-04107, Leipzig, Germany; 5 Max Planck Institute for Mathematics in the Sciences, Inselstraße 22 D-04103 Leipzig, Germany; 6 Fraunhofer Institute for Cell Therapy and Immunology, Perlickstraße 1, D-04103 Leipzig, Germany; 7 Institute for Theoretical Chemistry, University of Vienna, Währingerstraße 17, A-1090 Wien, Austria; 8 Center for non-coding RNA in Technology and Health, University of Copenhagen, Grønnegårdsvej 3, DK-1870 Frederiksberg C, Denmark; 9 Santa Fe Institute, 1399 Hyde Park Rd, Santa Fe, NM 87501, USA

## Abstract

**Background:**

Orthology analysis is an important part of data analysis in many areas of bioinformatics such as comparative genomics and molecular phylogenetics. The ever-increasing flood of sequence data, and hence the rapidly increasing number of genomes that can be compared simultaneously, calls for efficient software tools as brute-force approaches with quadratic memory requirements become infeasible in practise. The rapid pace at which new data become available, furthermore, makes it desirable to compute genome-wide orthology relations for a given dataset rather than relying on relations listed in databases.

**Results:**

The program Proteinortho described here is a stand-alone tool that is geared towards large datasets and makes use of distributed computing techniques when run on multi-core hardware. It implements an extended version of the reciprocal best alignment heuristic. We apply Proteinortho to compute orthologous proteins in the complete set of all 717 eubacterial genomes available at NCBI at the beginning of 2009. We identified thirty proteins present in 99% of all bacterial proteomes.

**Conclusions:**

Proteinortho significantly reduces the required amount of memory for orthology analysis compared to existing tools, allowing such computations to be performed on off-the-shelf hardware.

## Background

Genome annotation largely depends on the determination of sequence intervals that are homologous, and if possible, orthologous to sequences of known identity and function in related genomes. Orthologous genes (orthologs) are derived from a common ancestor by a speciation event [1]. Orthologs are of particular interest because they can be expected to have maintained at least part of their (ancestral) biological function. For protein-coding genes, several well-known databases, including InParanoid[2], OrthoMCL-DB[3], COG-database[4], Homogene[5], eggNOG[6], OMA Browser[7] and Ensembl Compara[8] compile such information. Their content is restricted to data *previously *published in comprehensive databases of protein sequences such as UniProt[9]. Updates with additional proteomic data thus are published relatively infrequently. Modern high-throughput technologies, however, produce huge amounts of protein data and even larger amounts of transcript data that are computationally translated to putative polypeptide sequences. Oftentimes, therefore, it would be desirable to generate the orthology relation for a particular dataset, so that the availability of orthology data does not limit the set of species or genes that can be included.

The computation of genome-wide orthology data, however, is a challenging and time consuming task with the currently available tools. In many cases, orthologs cannot be identified unambiguously by means of sequence comparison. The main difficulty arises from the presence of paralogs (homologous genes within the same genome) which can make it very difficult to recognize the correct ortholog among the other homologs. Gene duplications following the speciation, furthermore, create two or more genes in one lineage that are, collectively, orthologous to one or more genes in another lineage. Such genes are known as co-orthologs [10].

The most widely used approach to identify (putative) orthologs between two species is the *reciprocal best alignment heuristic *[11-15]. *This approach *was more recently extended e.g. in OrthoMCL[16] and MultiParanoid[17] to detect (co-)orthologs within multiple species. All these tools, however, are limited to relatively small sets of species. In practise, analyzing the complete proteomes of more than about 50 prokaryote species goes beyond the capabilities of standard hardware and requires access to supercomputer resources. This limitation arises from both, technical issues such as insufficient parallelization and the algorithmic design that requires all reasonable alignments for each input protein to be held in the memory for efficient access in the clustering stage. Proteinortho is specifically designed to deal with hundreds of species together containing millions of proteins. It achieves this performance both by optimizing the implementation and by modifying the reciprocal best alignment method in a way that allows alignment processing on the fly.

## Results and Discussion

### Orthology prediction

#### Theory

As for other approaches to large-scale orthology detection, the starting point is a complete collection of pairwise comparisons, typically performed using blast. For simplicity of presentation, we assume that the individual sequences that are compared represent proteins, although algorithms and pipelines are applicable also to other sequence data such as non-coding RNA genes or conserved DNA regions. Typically, the results of pairwise comparisons are ranked by similarity, for instance based on blast statistics, evolutionary distances, or genome rearrangement analysis [7,16,18]. High-ranking alignments across multiple species then have to be combined in order to determine orthologous groups. However, these groups usually do not readily provide detailed insights since they can contain large numbers of related genes for each species. Hence, meaningful units have to be identified. For this purpose, a variety of clustering algorithms has been applied to determine *Clusters of Orthologous Groups *(COGs). The MCL-algorithm for instance uses a stochastic flow simulation to determine meaningful COGs [16,19,20]. In addition, MultiParanoid explicitly searches and tags in-paralogs, i.e., recent paralogs that represent species-specific gene expansions. This strategy requires to directly compare proteins within each species. Alternatively, data were curated by manual postprocessing [4].

We will argue here that orthology determination can be understood as the problem of finding *nearly *disjoint maximal *nearly*-complete multipartite subgraphs in an edge-weighted directed graph  whose vertices are the proteins in the input set, and whose edges connect certain pairs of similar proteins of different species. The edge weights *ω*_*x*→*y *_encode the similarity of *x *and *y*. In our implementation, the bit score of the blast alignment (*x *→ *y*) will serve as edge weight. An *E*-value cut-off is used beyond which blast alignments are not included into .

**Figure 1:**
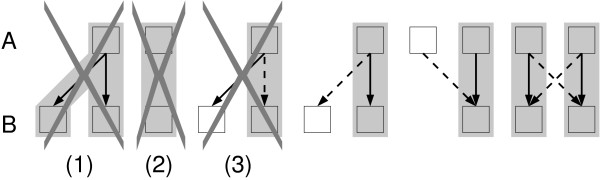
**Orthology relations**. Idealized dataset for two species *A *and *B*. Proteins *x *∈ *A *and *y *∈ *B *are depicted by open boxes. Orthology relations between proteins *x *and *y *are represented by grey shadows. Arrows indicate alignments above a certain cut-off from the search of *x *against *B*. Solid lines refer to the best alignments. Cases (1), (2), and (3) cannot occur by definition in an idealized dataset, but of course do appear in real life applications.

In order to motivate our point of view, we first consider an idealized dataset (see Figure 1) in which (1) each protein *x *(from species *A*) has at most one ortholog in any other species *B *≠ *A*, (2) if *y *∈ *B *is an ortholog of *x *∈ *A*, then a blast search of *x *against *B *yields at least one alignment and (3) the unique best alignment of query *x *against *B *is the true ortholog *y *of *x*. In this case, the well-know "reciprocal best alignment heuristic" (RBAH), also known as "reciprocal best hits" (RBH) [11], can be used to retrieve the correct ortholog set. To see this, we construct a subgraph  of  as follows: For each protein *x *in species *A *and a given species *B *≠ *A *we retain only the arc with maximal weight:(1)

The symmetric subgraph of , containing only reciprocal best alignments, can be regarded as an undirected graph ϒ*_RBAH_*. By construction, any two vertices are connected by edges in ϒ*_RBAH _*if and only if they are orthologs. A set of orthologs therefore corresponds to a complete multipartite subgraph of  in which every species is represented at most once. Furthermore, we note that these subgraphs are disjoint, i.e., ortholog sets correspond to the connected components of .

**Figure 2:**
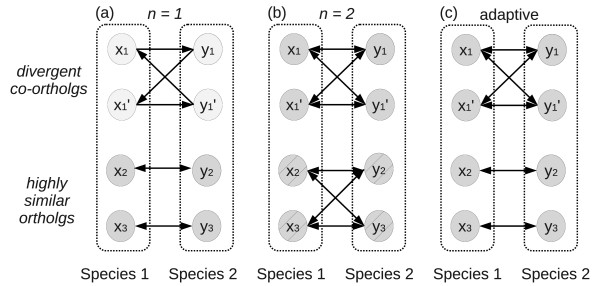
**Adaptive RBAH**. Reciprocal best alignment heuristic (RBAH). (a) If there is a pair of divergent co-orthologs *x*_1_, , and *y*_1 _and , resp., it is possible that there are no *reciprocal *best blast alignments. In this situation, RBAH will not identify any orthologs. (b) One possible remedy is to include the second best blast alignment (*n *= 2). However, in this case highly similar orthologs (*x*_2 _and *y*_2 _as well as *x*_3 _and *y*_3_), which in principle can clearly be divided, can get combined. (c) Proteinortho uses an adaptive approach that is (1) flexible with respect to the number of more diverged orthologs in absence of a reciprocal best blast alignment and (2) will not intermix orthologous groups that can be disentangled easily because of large differences in pairwise similarity.

When applied to real data, however, RBAH usually gives rise to several artifacts. In general, therefore, it does not produce correct and complete sets of orthologs. First, gene duplications produce co-orthologs, destroying the uniqueness of best blast alignments. The blast comparison of two species both containing two co-orthologs will in general produce slightly different scores among those genes, so that  in general will not contain all arcs between them, see Figure 2a. RBAH now extracts the symmetric part of , i.e., it removes all non-reciprocal edges. Thus the undirected graph ϒ*_RBAH _*has an edge {*x*, *y*} if and only if both (*x*, *y*) and (*y*, *x*) are arcs in . A missing arc in the example of Figure 2a thus translates into a missing edge in ϒ*_RBAH_*. Ortholog sets, therefore, are still located in disjoint connected components, but they are only approximately multipartite and there is no guarantee that they remain connected. As a remedy, it could be proposed to use the *k*-best blast alignments instead of a single best one. This leads, however, to another type of artifact: If distant homologs or spurious blast alignments with scores above the blast threshold are present, see Figure 2b, the inclusion of up to *k *alignments can result in edges between paralogous but non-orthologous proteins.

In order to reduce such problems, we suggest to introduce a similarity cut-off value that itself depends on the quality of the matches, i.e., to consider all those blast alignments of each query against a given species whose *E*-values are only slightly smaller (by a factor *f *< 1) than the queries' best blast alignments. More precisely,(2)

This has two advantages: it retains edges to likely co-orthologs while at the same time reducing the number of edges that are inserted in .

The symmetric part ϒ* of  now retains more edges than ϒ*_RBAH_*. In particular, it includes all the edges connecting similar co-orthologs. On the other hand, the threshold at fairly high bit scores disconnects at least most of the more distant homologs. Sets of (co-)orthologs thus appear in ϒ* as nearly complete multipartite subgraphs. Typically they will contain more than one node from the same species, among them, in particular, all in-paralogs. Although this approach strongly reduces the problem with spurious edges, ϒ* may also contain additional edges connecting two or more sets of (co-)orthologs.

The problem of finding maximal complete multipartite subgraphs of a graph is NP complete [21]. Furthermore, we have seen above that ϒ* may lack a few edges which should connect orthologs ("false negatives"), while at the same time there are also some additional "false positive" edges. In a two-species comparison there is no information that could compensate missing and spurious edges, while in the multi-species case, the graph ϒ* is in a sense "self-correcting" since we can formulate orthology detection as an optimization problem. More precisely, we search for a decomposition of ϒ* into a disjoint collection of complete multipartite subgraphs so that the number of edge insertions and deletions is minimized.

Since no efficient approaches to this combinatorial optimization problem seem to be known, it appears fruitful to resort to a heuristic approach that employs a somewhat different point of view: nearly complete multipartite subgraphs are very dense subgraphs, which in our case either form connected components on their own, or which are connected to other dense clusters by a few additional edges. The problem thus is to determine for each connected component ϒ* of whether it is sufficiently densely connected, and if not, to partition it into its densely connected components by removing the spurious edges connecting them. Here, we approach this issue by means of spectral partitioning [22], see Additional File 1 for a detailed description.

We remark, finally, that one could efficiently add the explicit determination of in-paralogs after ϒ* has been constructed, although currently this is not implemented in Proteinortho. Following e.g. InParanoid, sets of in-paralogs are subsets of proteins from the same species within the same connected component ϒ* of that are more similar to each other than to any protein in another species. It is sufficient, thus, to determine alignment scores for pairs of nodes from the same species within connected components of ϒ*. As in MultiParanoid, in-paralogs could be collapsed to a single node.

#### Implementation

##### Blast searches

**Figure 3:**
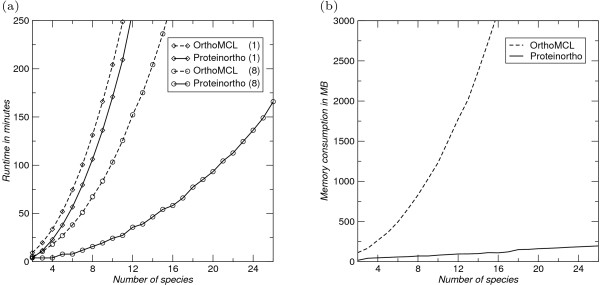
**Benchmarks**. CPU time and memory requirements of Proteinortho. (a) The speed benchmark was performed using an *E. coli *strain with 4132 proteins on an eight core Intel Xeon system using one thread (1) and eight threads (8) at 2.33 GHz. The encoded proteins were used multiple times to simulate multiple (identical) species. This is the worst case scenario for Proteinortho since in this case every protein has a link to at least one protein in every other species. Proteinortho is significantly faster than OrthoMCL. Using multiple threads we observe a substantial speed up. (b) The memory benchmark is performed using the same set as in (a). OrthoMCL quickly exhausts memory for larger sets. Proteinortho clearly performs more efficient, even though this artificial scenario is a more complex case than real world analysis. Both benchmarks outline that Proteinortho allows comprehensive studies which were not possible before.

Proteinortho expects fasta files containing either nucleic acid or amino acid sequences as input. Proteinortho does not build a large database containing all proteins but rather keeps the protein complements of different species separate. This has multiple advantages: (1) the blast step can be partitioned into multiple runs. The available processor cores are used efficiently as each blast process can utilize one processor core to the full, which is usually not the case if threading is handled by blast itself. Furthermore, the blast jobs can be distributed to several computers in a network. (2) The computationally expensive blast search of species against themselves (which is implicit in searching a comprehensive protein database) can be avoided. (3) The *E*-values returned by blast depend only on the proteome of each input species, not on the database of all input sequences. Therefore, Proteinortho implicitly enforces higher similarity for related proteomes but allows lower levels of similarity for larger evolutionary distances. Conceptually, this replaces the normalization of the scores employed e.g. by OrthoMCL. (4) The scope of the analysis can be extended without the need to re-compute blast comparisons that have already been computed earlier. The *E*-values remain unchanged when species are included or excluded from the analysis. Proteinortho can use several PCs with a shared storage (such as an NFS file system). The implementation of distributed computing is illustrated in Additional File 2. Furthermore, cluster infrastructure (e.g. MPI or SGE) is supported. A benchmark illustrating the performance improvements is shown in Figure 3a. Proteinortho is faster than OrthoMCL even if only a single processor core is available and gains much of its practical advantage from parallelization. In applications to large numbers of species, for which Proteinortho is primarily intended, handling blast alignments dominates the spectral partitioning step by several orders of magnitude. As blast and the memory consumption of holding the graph structure for clustering are limiting, we do not investigate the complexity of the clustering step itself in detail.

Most importantly, the algorithm outlined in the previous section avoids the memory bottleneck that limits previous approaches. Suppose our input set comprises *N *species with, on average, *m *genes. The size of the input is thus *n *= *N *× *m *proteins. Instead of storing all *n *× *n *pairwise blast scores, Proteinortho processes the comparisons between any two species *A *and *B *immediately: first the blast alignments are filtered by two additional criteria: (1) The alignment must exhibit a minimum level of sequence identity. (2) The alignment must cover at least a minimum fraction of the query protein. This second rule ensures that fusion genes such as rice OsUK/UPRT1 [23] are eventually assigned as homologs of the dominating part of the protein. Then equ.(2) is evaluated for all *x *in *A*, so that Proteinortho directly constructs the sparse graph , while  does not need to be stored at all. Proteinortho therefore uses chained arrays, requiring only *n *× *k *entries, where *k *is the average number of nearly optimal blast alignments per gene, and *k *= *a *× *N*, where *a *is the average number of (co-)orthologs of a gene in a single species. The value of *a *is independent of the size of the dataset. Empirically, we found *a *≤ 1 in all datasets investigated so far. Thus Proteinortho saves a factor *n*^2^/*N*^2^*ma *= *m*/*a *≥ *m *of memory. Note that prokaryotes have *m *≈ 10^3 ^... 10^4 ^proteins.

##### Spectral partitioning

First we reduce the problem by determining the connected components ϒ* of since these can be treated separately. We use the well-known breath-first search approach [24] to this end. In order to check whether a connected component Ξ is sufficiently dense to represent a single set of co-orthologs we compute its normalized algebraic connectivity . Here *n *is the number of vertices of Ξ and *αalpha*_2 _is 2nd-smallest eigenvalue of the graph Laplacian **L **= **D **- **A **of Ξ [25]. Here **A **is the adjacency matrix of Ξ and **D **is the diagonal matrix of the vertex degrees. The eigenvalue *αalpha*_2 _can be computed iteratively, see Additional File 1. Values of  indicate dense clusters that most likely correspond to coherent sets of (co-)orthologs. Small values , on the other hand indicate that Ξ has a low connectivity and either consists of two or more dense components or it has (nearly) tree-like protrusions. Very large components  can arise when genes duplicate frequently and diverge quickly according to the duplication-degeneration-complementation (DDC) model [26].

The "Fiedler vector" **x**_2_, i.e., the eigenvector of **L **to eigenvalue *α*_2 _can be used to find a partition of Ξ into two connected components, one consisting of the vertices for which **x**_2 _has positive entries and one for which **x**_2 _has negative entries [27]. This decomposition is iterated until Ξ is partitioned into components with algebraic connectivity  above a certain threshold value and tree-like pieces, which most likely correspond to false-positive edges of ϒ*. In order to speed up the computation, trees are therefore removed from the component Ξ before the algebraic connectivity and the Fiedler vector is computed. This is achieved by iteratively removing a vertex of degree 1 and its adjacent edge. This step is not performed if Proteinortho is used to compare only two species.

We remark that the memory and CPU consumption for the clustering step of OrthoMCL can be drastically reduced by using a novel algorithm [28], reaching a performance that is theoretically comparable to spectral partitioning as used by Proteinortho (see Additional File 1). Both require only the storage of edge or adjacency lists. The current implementation of spectral partitioning could be further optimized e.g. by employing the Lanczos algorithm [29] for computing the eigenvalues. Spectral partitioning on average scales as *O*(*n*^2^*k*). This leads to an expected runtime of *O*(*N*^3^) for Proteinortho which is comparable to the *O*(*N*^3 ^log *N*) complexity bound achieved for COG clustering in [28].

### **Evaluation of **Proteinortho

We compared Proteinortho with the COG-database [4] and OrthoMCL[3]. The latter is the main competitor in terms of speed and memory. The COG-database provides a manually curated dataset that can be regarded as more reliable than fully automated approaches. For benchmark analysis, a set of 16 randomly chosen bacteria from three different classes (six Gram-positive bacilli, six gamma- and four alpha-proteobacteria) are used. The input set comprises 53, 623 protein sequences.

**Figure 4:**
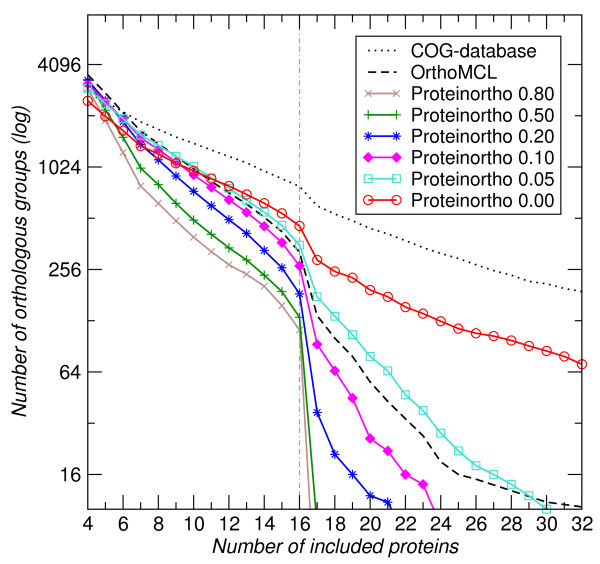
**Coverage completion**. Comparison of the results of Proteinortho with different thresholds of the normalized algebraic connectivity  with the COG-database and OrthoMCL for a dataset consisting of 16 randomly chosen bacterial proteomes. The vertical dashed line marks the transition from clusters containing mainly a single ortholog from each species to sets including co-orthologs. The COG-database reports many large groups which often include co-orthologous proteins. OrthoMCL and Proteinortho focus on highly connected subsets in order to find orthologous sets and thus split those groups. Thereby, Proteinortho's clustering algorithm becomes more stringent with increasing values of  in splitting in particular large groups. While these groups are left intact for , thresholds of 0.5 and higher drastically reduce the fraction of included co-orthologs.

Figure 4 summarizes the size distribution of co-ortholog sets for the three approaches. Using default settings (*E*-value cutoff 10^-10^, algebraic connectivity threshold , minimum 25% identity, 50% coverage of query sequence, similarity threshold *f *= 0.95) Both Proteinortho and OrthoMCL report fewer groups than listed in the COG-database. This is not surprising, however, since the COG-database relies on less restrictive criteria and thus tends to include multiple co-orthologs, while both stand-alone tools apply clustering algorithms that attempt to split the large components whenever possible.

**Figure 5:**
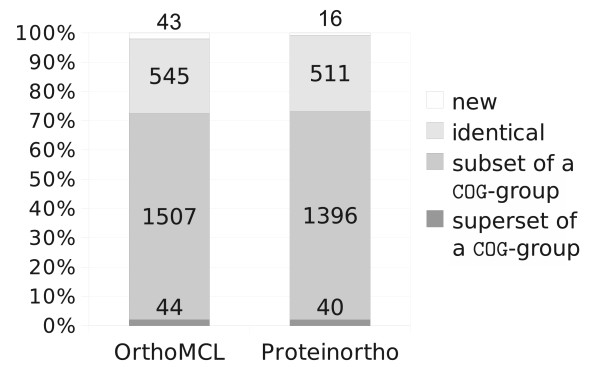
**Comparison of results**. Comparison of OrthoMCL and Proteinortho to the COG-database. The following assignments were defined: identity: the group equals a COG-group; subset: the group is subset of a COG-group, at least two proteins are equal; superset: the group is a superset of a COG-group, at least two proteins are equal; new: none of the above-noted criteria matched. Both tools reveal comparable results with respect to the manually curated COG-database. OrthoMCL covers more identical and differently composed groups while Proteinortho is more restrictive and reports fewer new groups which are not present in the COG-database. All groups with less than six species were omitted from the OrthoMCL and Proteinortho data. See Additional File 2 for comparisons with different minimal coverage.

Figure 5 presents the outcome in more detail. Proteinortho and OrthoMCL report comparable results. Proteinortho is more stringent. The amount of completely new groups which have to be regarded as false positives is considerably lower. OrthoMCL reports slightly more groups. Even though both tools were applied with the same E-value threshold the results vary due to the different blast strategies: OrthoMCL employs one large database containing all proteins, while Proteinortho uses species-specific databases. Most pairwise *E*-values derived by Proteinortho thus tend to be somewhat larger than those derived by OrthoMCL for the same set of proteins. In turn, OrthoMCL reports some more and larger groups. However, the overall outcome can be regarded as similar while the runtime and memory requirements of Proteinortho are substantially reduced in comparison to OrthoMCL.

### Domain-wide commons

In order to demonstrate that Proteinortho is suitable for large-scale analysis we asked *Which proteins can be found in all bacterial species? *Proteins that are conserved domain-wide are likely to be useful for the construction of a phylogeny of eubacteria as an alternative to the prevalent usage of 16*S *rRNA sequences [30]. They can also serve as protein-based markers for identifying novel bacterial species as members of an established phylogenetic group. In addition, they can give insight into basic protein equipment of bacterial life. Hence, we applied Proteinortho to the set of all eubacterial proteomes available at NCBI at the beginning of 2009 (Additional File 3).

The input dataset comprises 2, 155, 620 proteins annotated in 717 bacterial genomes. The Proteinortho run took less than two weeks using 50 processor cores (Intel Xeon at 2.00-2.33 GHz) distributed over multiple PCs. Only 2 GB memory were required. OrthoMCL could not be employed for this task on the hardware available in our lab. Extrapolating from the benchmarks in Figure 3, we estimate that hundreds of gigabytes of memory and years of runtime would have been required.

Proteinortho identified 152 proteins as core of the bacterial protein complement, occurring in at least 90% of all 717 free-living and endosymbiotic bacteria. Of these, 32 are ribosomal subunits. The 30 apparently most indispensable proteins, occurring in 99% of all bacteria, are:

Elongation factor Tu (often co-orthologous to elongation factor 1-alpha)

Elongation factor G

Translation initiation factor IF-2

RNA polymerase subunits *β *and *β'*

ATP-dependent metalloprotease FtsH

O-sialoglycoprotein endopeptidase

Methionine aminopeptidase

F0F1 ATP synthase subunits *α *and *β*

Dimethyladenosine transferase

1 ribosomal protein of the *30S *rRNA subunit

3 ribosomal proteins of the *50S *rRNA subunit

3 GTP-binding proteins

12 tRNA synthetases

**Figure 6:**
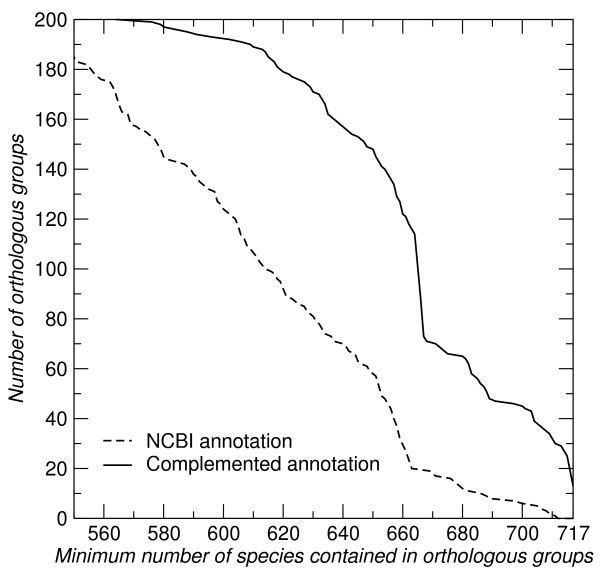
**Orthologous groups**. Number of orthologous groups present in nearly all bacterial species. The dashed line represents Proteinortho results based on the NCBI annotation. Using tblastn the annotation was complemented with high scoring genomic matches (solid curve). Note that this is a cumulative plot, i.e., each group of co-orthologs present in *x *species is also included in the count of groups contained in *x*' <*x *species.

A more detailed list, including unique identifiers for groups of proteins can be found in the supplemental material. The sensitivity of Proteinortho in this survey is limited by incomplete annotation in many species, which we complemented here by tblastn searches with fairly restrictive cut-off values, see Methods for details. Figure 6 summarizes the results quantitatively, emphasizing the effect of incomplete annotation. We remark that this example also shows that Proteinortho can be used to complement existing annotations in an automatic fashion.

Nevertheless, about one third of the 30 most conserved proteins could not be recovered in the genomes of the two species with the smallest proteomes in our dataset: *Candidatus Carsonella ruddii PV *and *Candidatus Sulcia muelleri GWSS*. Both are endosymbionts that are considered as organelle-like [31,32]. Numerous genes that are otherwise considered to be essential for life have been reported as missing in both species. A more detailed and larger list of domain-wide common proteins can be downloaded at http://bioinf.pharmazie.uni-marburg.de/supplements/proteinortho/.

## Conclusions

Proteinortho implements a blast-based approach to determine sets of (co-)orthologous proteins or nucleic acid sequences that generalizes the reciprocal best alignment heuristic. The software is optimized for large datasets, and in particular provides a drastic reduction of the memory requirements compared to earlier tools. It can therefore be run on off-the-shelf PC hardware for large datasets. Our implementation scales very well with the number of available processor cores. The blast searches can be trivially parallelized and distributed easily to multiple PCs without the need for a cluster management system, while deployment to existing cluster infrastructure is also supported.

Proteinortho views orthology detection as a variant of graph clustering since co-orthologous sets correspond to maximal complete multipartite subgraphs, which at the same time are well separated from each other. Due to the unavoidable noise in the real data, however, co-orthologous sets appear as dense subgraphs without clearly recognizable low-weight cuts. This property is measured quite well by the algebraic connectivity. At the same time, low-weight cuts between dense regions are identified very well by the corresponding Fiedler vector. We therefore employ spectral partitioning instead of a direct graph clustering approach. The quality of the co-orthologous sets proposed by Proteinortho is comparable to the performance of OrthoMCL.

Both time and memory requirements are significantly reduced compared to earlier approaches, enabling applications that were infeasible before. For instance, we applied Proteinortho to the complete set of 2.1 million proteins from the 717 bacterial genomes available at NCBI at the beginning of 2009. We found 30 proteins that are present in more than 99% of the investigated sequences.

## Methods

All analysis with Proteinortho and OrthoMCL were applied using default values unless described otherwise. These are *E *- *value *< 10^-10^, 25% percent identity, Markov Inflation Index of 1.5 for OrthoMCL and *E*-value < 10^-10^, 25% percent identity, adaptive best alignments similarity of *f *= 0.95, algebraic connectivity > 0.1 for Proteinortho. OrthoMCL version 1.4 was downloaded from http://OrthoMCL.org/common/downloads/.

Speed and memory benchmark were performed multiple times using the proteome of *Escherichia coli K12 substr. MG1655 *data from the NCBI. The protein ids were renamed systematically to prevent duplicated ids for benchmarking purposes which cannot be handled by Proteinortho. A script continuously observed the memory consumption and reported the maximum peak for each run, Figure 3.

For the domain-wide commons we applied Proteinortho with default values of the parameters. Bacterial proteomes and genomes were downloaded from NCBI ftp://ftp.ncbi.nih.gov/genomes/Bacteria/ in March 2009. A detailed list can be found in Additional File 3. In order to recover missing annotation, we selected all orthologous groups covering at least 75% of all species that are good candidates of domain-wide commons. The unique set of sequences of each orthologous group was blasted against all genomes that lack an annotated ortholog using tblastn (*E*-value < 10^-20^). The sequence of the best alignment was then added to the orthologous group.

For evaluation we used the proteome data from the COG-database ftp://ftp.ncbi.nih.gov/pub/COG/COG/ downloaded in November 2009. We have chosen *Bacillus halodurans, Bacillus subtilis, Lactococcus lactis, Listeria innocua, Streptococcus pneumoniae TIGR4, Streptococcus pyogenes M1 GAS *from the Gram-positive bacilli class, *Buchnera sp. APS, Escherichia coli K12, Pasteurella multocida, Salmonella typhimurium LT2, Vibrio cholerae, Yersinia pestis *from the gamma proteobacteria class and *Brucella melitensis, Caulobacter vibrioides, Mesorhizobium loti, Rickettsia prowazekii *from the alpha proteobacteria class. Both, Proteinortho and OrthoMCL were applied to this set. All groups with proteins covering at least 6 species were compared to the COG-database, illustrated in Figure 4 and Figure 5.

## Availability

The source code of Proteinortho can be obtained under the GPLv2 (or later) from http://www.bioinf.uni-leipzig.de/Software/proteinortho/

## Authors' contributions

SP implemented an initial RBAH prototype. ML implemented, optimized, and evaluated the later versions of the program. All authors contributed to the details of implementation, testing and interpretation of the data, and collaborated in writing and approved the final manuscript.

## Supplementary Material

Additional File 1 **Algebraic connectivity and Fiedler vector**. Iterative approximation of the Algebraic Connectivity using the Fiedler Vector. Click here for file

Additional File 2 **Supplemental figures**. Supplemental Figures showing how multiple instances of Proteinortho can cooperate and how the comparison to OrthoMCL looks using smaller cutoffs. Click here for file

Additional File 3 **Species list for domain wide commons**. Table of species and accession numbers used in this analysis. Click here for file
