# Supplementary material for: Proteinortho: Detection of (Co-)orthologs in large-scale analysis
Source: BMC Bioinformatics. 2011 Apr 28;12:124. doi: 10.1186/1471-2105-12-124 (PMC3114741; doi:10.1186/1471-2105-12-124)
Supplement: Additional File 1 — Algebraic connectivity and Fiedler vector. Iterative approximation of the Algebraic Connectivity using the Fiedler Vector. [file 1471-2105-12-124-S1.PDF]

Algebraic Connectivity and Fiedler Vector

Consider an undirected graph Γ with n vertices. Its adjacency matrix $\mathbf{A}$ has the entries $A_{xy} = 1$ if $\{x, y\}$ is an edge in Γ, and $A_{xy} = 0$ otherwise. The matrix $\mathbf{D}$ has the diagonal entries $D_{xx} = \sum_y A_{xy}$ for all vertices x and vanishing off-diagonal elements $D_{xy} = 0$ if $x \neq y$. The graph Laplacian of Γ is the matrix $\mathbf{L} = \mathbf{D} - \mathbf{A}$, see [1].

We recall that the vector $\vec{1} = (1, \ldots, 1)$ is an eigenvector of $\mathbf{L}$ with eigenvalue 0. Since $\mathbf{L}$ is non-negative definite, 0 is the smallest eigenvalue of $\mathbf{L}$. It is simple if and only Γ is connected. The 2nd smallest eigenvalue of $\mathbf{L}$ is the so-called *algebraic connectivity* of Γ. If is strictly positive whenever Γ is connected. It is bounded from above by the vertex connectivity of Γ [2]. The associated eigenvector $\mathbf{x_2}$ in known as Fiedler vector. Its weak nodal domains, i.e., the subgraph induced by the vertices with non-negative entries in $\mathbf{x_2}$ and the subgraph induced by the vertices with non-negative entries in $\mathbf{x_2}$ are connected [3]. Hence α_2 provides a good measure to determine whether Γ is dense (when α_2 is close to n, the maximally possible value), while the Fiedler vector can be used to partition graphs with small α_2 into two subgraphs corresponding to the two nodal domains.

In order to get a numerically stable iteration for α_2, we need to transform $\mathbf{L}$ so that $\vec{1}$ is not the smallest but the largest eigenvalue also in absolute value. The largest eigenvalue of $\mathbf{L}$ is not larger than

$$2 \max_x D_x x = 2\Delta$$

The auxiliary matrix

$$\mathbf{Q} = (2\Delta + 1)\mathbf{I} - \mathbf{L} = (2\Delta + 1)\mathbf{I} - \mathbf{D} + \mathbf{A}$$

therefore has the desired properties. Its largest eigenvalue is $2\Delta + 1$ with eigenvector $\vec{1}$ and its 2nd largest eigenvalue is $\lambda_2 = 2\Delta + 1 - \alpha_2$. To calculate λ_2, we start with an arbitrary vector $\vec{x}$. Its coordinates are taken from a random number generator. From the vector, we first construct $\hat{x}$ by subtracting the average from each coordinate.

$$\hat{x} = \vec{x} - \frac{1}{n} \sum_{i=1}^{n} x_i$$

Then we normalize $\hat{x}$ by dividing each coordinate by its length.

$$\hat{x}^* = (1/\|\hat{x}\|)\hat{x}$$

The resulting vector $\hat{x}^*$ has unit length and is orthogonal to $\vec{1}$. Now we compute $\vec{y} = \mathbf{Q}\hat{x}^*$. Component-wise we have:

$$y_i = (\mathbf{Q}\hat{x}^*)_i = (2\Delta - d_i)\hat{x}_i^* + (\mathbf{A}\hat{x}^*)_i$$

where the latter term is computed directly via summing over the entries of $\hat{x}^*$ indexed by the chained arrays which represent the adjacency list $l_i = [j_1, \ldots, j_{d_i}]$

$$(\mathbf{A}\hat{x}^*)_i = \sum_{j=1}^{n} A_{ij}\hat{x}^*_j = \sum_{k=1}^{d_i} \hat{x}^*_{j_k}$$

Thus, $\mathbf{A}\hat{x}^*$ an be calculated in $\mathcal{O}(M)$ time and space, where M is the number of edges of G. Although $\vec{y}$ is theoretically already orthogonal to $\vec{1}$, we subtract the projection onto $\vec{1}$ in each iteration to stabilize the computation against round-off errors. Finally, we compute its length $\|\hat{y}\|$ which converges to λ_2. Hence, we record $\|\hat{y}\|$, $\hat{x}^*$ is replaced by $\frac{1}{\|\hat{y}\|}\hat{y}$ and the the computation of $\|\hat{y}\|$ is repeated until $\|\hat{y}\|$ and $\hat{x}^*$ do not change more than some prescribed accuracy bound. The vector $\hat{x}^*$ then has converged to the Fiedler vector $\mathbf{x_2}$.

Finally, we compute the algebraic connectivity as $\alpha_2 = 2\Delta + 1 - \lambda_2$ and normalized algebraic connectivity is set to $\alpha_2^* = \frac{\alpha_2}{n}$.

References

[1] Bıyıkoğlu T, Leydold J, Stadler PF: *Laplacian Eigenvectors of Graphs: Perron-Frobenius and Faber-Krahn Type Theorems*, *Volume 1915 of* Lecture Notes in Mathematics. Heidelberg: Springer Verlag 2007.

[2] Fiedler M: **Algebraic Connectivity of Graphs**. *Czechoslovak Math. J.* 1973, **23**:298–305.

[3] Fiedler M: **A property of eigenvectors of nonnegative symmetric matrices and its application to graph theory**. *Czechoslovak Math. J.* 1975, **25**:619–633.
