# Supplementary material for: Proteinortho: Detection of (Co-)orthologs in large-scale analysis
Source: BMC Bioinformatics. 2011 Apr 28;12:124. doi: 10.1186/1471-2105-12-124 (PMC3114741; doi:10.1186/1471-2105-12-124)
Supplement: Additional File 2 — Supplemental figures. Supplemental Figures showing how multiple instances of Proteinortho can cooperate and how the comparison to OrthoMCL looks using smaller cutoffs. [file 1471-2105-12-124-S2.PDF]

Supplemental Figures

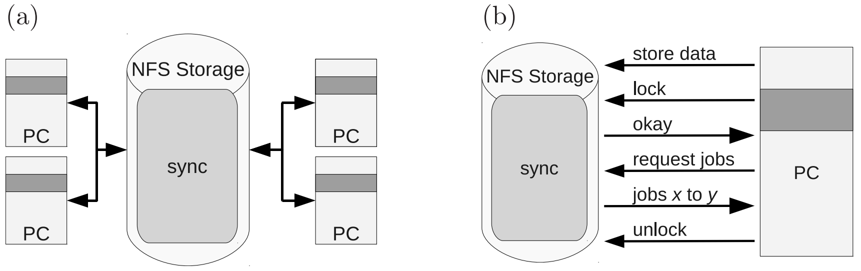

Figure 1: `Proteinortho` can use multiple PCs straight away. A cluster management system is not necessary.
(a) Multiple PCs running `Proteinortho`. They cooperate dynamically using a N-way technique. All `blast` jobs are distributed onto individual PCs. The results are stored on a shared NFS storage. Coordination of jobs is done via a synchronization file (sync).
(b) Communication of a single PC with the storage. Whenever a job is finished the results are stored, the synchronization file is locked and new jobs are chosen according to it. This information is then available to other PCs requesting new jobs. The file gets unlocked afterwards.

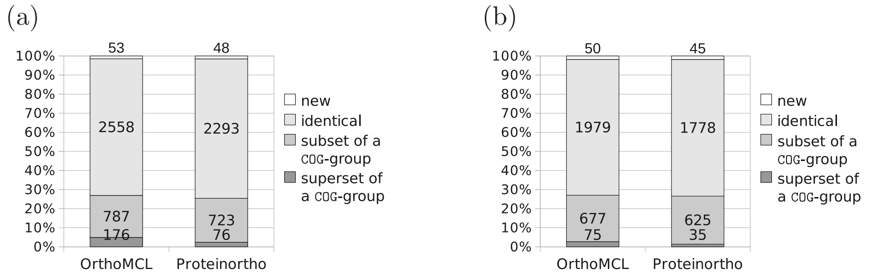

Figure 2: Comparison of `OrthoMCL` and `Proteinortho` to the `COG`-database. The following assignments were defined: identity: the group equals a `COG`-group; subset: the group is subset of a `COG`-group, at least two proteins are equal; superset: the group is a superset of a `COG`-group, at least two proteins are equal; new: none of the above-noted criteria matched. Both tools reveal comparable results with respect to the manually curated `COG`-database. `OrthoMCL` covers more identical and differently composed groups while `Proteinortho` is more restrictive and reports fewer new groups which are not present in the `COG`-database. (a) All groups with less than four species were dismissed from the `OrthoMCL` and `Proteinortho` data.
(b) All groups with less than five species were dismissed from the `OrthoMCL` and `Proteinortho` data.
