# Supplementary material for: Proteinortho: Detection of (Co-)orthologs in large-scale analysis
Source: BMC Bioinformatics. 2011 Apr 28;12:124. doi: 10.1186/1471-2105-12-124 (PMC3114741; doi:10.1186/1471-2105-12-124)
Supplement: Additional File 3 — Species list for domain wide commons. Table of species and accession numbers used in this analysis. [file 1471-2105-12-124-S3.PDF]

Species list for domain wide commons

Species	Accession number(s)	Last updated
Acaryochloris marina MBIC11017	NC_009925	2007/11/20
Acholeplasma laidlawii PG 8A	NC_010163	2008/03/18
Acidiphilium cryptum JF-5	NC_009484	2007/05/23
Acidithiobacillus ferrooxidans ATCC 53993	NC_011206	2008/09/08
Acidobacteria bacterium Ellin345	NC_008009	2007/01/23
Acidothermus cellulolyticus 11B	NC_008578	2007/01/23
Acidovorax avenae citrulli AAC00-1	NC_008752	2007/01/05
Acidovorax JS42	NC_008782	2007/01/11
Acinetobacter baumannii AB0057	NC_011586	2008/11/18
Acinetobacter baumannii ACICU	NC_010611	2008/06/11
Acinetobacter baumannii ATCC 17978	NC_009085	2007/03/07
Acinetobacter baumannii AYE	NC_010410	2008/03/19
Acinetobacter baumannii SDF	NC_010400	2008/03/14
Acinetobacter sp ADP1	NC_005966	2007/01/23
Actinobacillus pleuropneumoniae L20	NC_009053	2007/02/26
Actinobacillus pleuropneumoniae serovar 3 JL03	NC_010278	2008/01/24
Actinobacillus pleuropneumoniae serovar 7 AP76	NC_010939	2008/06/13
Actinobacillus succinogenes 130Z	NC_009655	2007/07/25
Aeromonas hydrophila ATCC 7966	NC_008570	2007/01/23
Aeromonas salmonicida A449	NC_009348	2007/04/18
Aeropyrum pernix	NC_000854	2007/01/23
Akkermansia muciniphila ATCC BAA 835	NC_010655	2008/07/27
Alcanivorax borkumensis SK2	NC_008260	2008/01/07
Aliivibrio salmonicida LFI1238	NC_011312, NC_011313	2008/10/21
Alkalilimnicola ehrlichei MLHE-1	NC_008340	2007/01/23
Alkaliphilus metalliredigens QYMF	NC_009633	2007/07/03
Alkaliphilus oremlandii OhILAs	NC_009922	2007/10/17
Alteromonas macleodii Deep ecotype	NC_011138	2008/08/15
Anabaena variabilis ATCC 29413	NC_007413	2007/01/23
Anaeromyxobacter dehalogenans 2CP-C	NC_007760	2007/01/23
Anaeromyxobacter Fw109-5	NC_009675	2007/07/25
Anaeromyxobacter K	NC_011145	2008/08/23
Anaplasma marginale St Maries	NC_004842	2007/04/25
Anaplasma phagocytophilum HZ	NC_007797	2007/01/23
Anoxybacillus flavithermus WK1	NC_011567	2008/11/14
Aquifex aeolicus	NC_000918	2005/12/04
Archaeoglobus fulgidus	NC_000917	2007/01/23
Arcobacter butzleri RM4018	NC_009850	2007/09/29
Arthrobacter aurescens TC1	NC_008711	2006/12/28
Aster yellows witches-broom phytoplasma AYWB	NC_007716	2006/01/19
Azoarcus BH72	NC_008702	2008/01/07
Azoarcus sp EbN1	NC_006513	2007/01/23
Azorhizobium caulinodans ORS 571	NC_009937	2007/10/19
Bacillus amyloliquefaciens FZB42	NC_009725	2007/08/04
Bacillus anthracis Ames 0581	NC_007530	2007/01/23
Bacillus anthracis Ames	NC_003997	2007/01/23
Bacillus anthracis str Sterne	NC_005945	2005/12/04
Bacillus cereus ATCC 10987	NC_003909	2007/01/23
Bacillus cereus ATCC14579	NC_004722	2005/12/04
Bacillus cereus cytotoxis NVH 391-98	NC_009674	2007/07/25
Bacillus cereus ZK	NC_006274	2007/01/23
Bacillus clausii KSM-K16	NC_006582	2007/01/23
Bacillus halodurans	NC_002570	2007/01/23

Bacillus licheniformis ATCC 14580	NC_006270	2007/12/26
Bacillus licheniformis DSM 13	NC_006322	2007/12/26
Bacillus pumilus SAFR-032	NC_009848	2007/09/27
Bacillus subtilis	NC_000964	2008/02/19
Bacillus thuringiensis Al Hakam	NC_008600	2007/01/24
Bacillus thuringiensis konkukian	NC_005957	2005/12/04
Bacillus weihenstephanensis KBAB4	NC_010184	2008/03/18
Bacteroides fragilis NCTC 9434	NC_003228	2007/01/23
Bacteroides fragilis YCH46	NC_006347	2005/12/04
Bacteroides thetaiotaomicron VPI-5482	NC_004663	2005/12/04
Bacteroides vulgatus ATCC 8482	NC_009614	2007/06/29
Bartonella bacilliformis KC583	NC_008783	2007/01/11
Bartonella henselae Houston-1	NC_005956	2005/12/04
Bartonella quintana Toulouse	NC_005955	2005/12/04
Bartonella tribocorum CIP 105476	NC_010161	2007/12/26
Baumannia cicadellinicola Homalodisca coagulata	NC_007984	2006/05/08
Bdellovibrio bacteriovorus	NC_005363	2007/01/23
Beijerinckia indica ATCC 9039	NC_010581	2008/04/12
Bifidobacterium adolescentis ATCC 15703	NC_008618	2006/12/08
Bifidobacterium longum DJO10A	NC_010816	2008/06/06
Bifidobacterium longum infantis ATCC 15697	NC_011593	2008/11/22
Bifidobacterium longum	NC_004307	2007/01/23
Bordetella avium 197N	NC_010645	2008/05/07
Bordetella bronchiseptica	NC_002927	2007/01/23
Bordetella parapertussis	NC_002928	2007/01/23
Bordetella pertussis	NC_002929	2007/01/23
Bordetella petrii	NC_010170	2008/02/08
Borrelia afzelii PKo	NC_008277	2007/01/24
Borrelia burgdorferi	NC_001318	2007/06/13
Borrelia duttonii Ly	NC_011229	2008/09/18
Borrelia hermsii DAH	NC_010673	2008/09/11
Borrelia recurrentis A1	NC_011244	2008/09/18
Borrelia turicatae 91E135	NC_008710	2008/07/25
Bradyrhizobium BTAi1	NC_009485	2007/05/23
Bradyrhizobium japonicum	NC_004463	2005/12/04
Bradyrhizobium ORS278	NC_009445	2008/01/07
Buchnera aphidicola Cc Cinara cedri	NC_008513	2007/01/23
Buchnera aphidicola	NC_004545	2005/12/04
Buchnera aphidicola Sg	NC_004061	2005/12/04
Buchnera sp	NC_002528	2007/01/23
Burkholderia cenocepacia J2315	NC_011000, NC_011001, NC_011002	2008/09/24
Burkholderia xenovorans LB400	NC_007952, NC_007953	2008/09/10
Caldicellulosiruptor saccharolyticus DSM 8903	NC_009437	2007/05/08
Caldivirga maquilingensis IC-167	NC_009954	2007/11/08
Campylobacter concisus 13826	NC_009802	2007/09/14
Campylobacter curvus 525 92	NC_009715	2007/07/31
Campylobacter fetus 82-40	NC_008599	2007/01/24
Campylobacter hominis ATCC BAA-381	NC_009714	2007/07/31
Campylobacter jejuni 81116	NC_009839	2007/09/22
Campylobacter jejuni 81-176	NC_008787	2007/01/11
Campylobacter jejuni doylei 269 97	NC_009707	2007/07/27
Campylobacter jejuni	NC_002163	2005/12/04
Campylobacter jejuni RM1221	NC_003912	2007/01/23
Candidatus Amoebophilus asiaticus 5a2	NC_010830	2008/07/15
Candidatus Azobacteroides pseudotrichonymphae genomovar CFP2	NC_011565	2008/11/15

Candidatus Blochmannia floridanus	NC_005061	2005/12/04
Candidatus Blochmannia pennsylvanicus BPEN	NC_007292	2007/01/23
Candidatus Carsonella ruddii PV	NC_008512	2007/01/23
Candidatus Desulfococcus oleovorans Hxd3	NC_009943	2007/10/23
Candidatus Desulforudis audaxviator MP104C	NC_010424	2008/03/18
Candidatus Korarchaeum cryptofilum OPF8	NC_010482	2008/03/19
Candidatus Methanoregula boonei 6A8	NC_009712	2007/07/31
Candidatus Pelagibacter ubique HTCC1062	NC_007205	2005/12/04
Candidatus Phytoplasma australiense	NC_010544	2008/08/27
Candidatus Phytoplasma mali	NC_011047	2008/07/15
Candidatus Ruthia magnifica Cm Calyptogena magnifica	NC_008610	2007/01/23
Candidatus Sulcia muelleri GWSS	NC_010118	2007/12/10
Candidatus Vesicomyosocius okutanii HA	NC_009465	2007/05/23
Carboxydothermus hydrogenoformans Z-2901	NC_007503	2007/01/23
Caulobacter crescentus	NC_002696	2005/12/04
Caulobacter K31	NC_010338	2008/03/18
Cellvibrio japonicus Ueda107	NC_010995	2008/06/24
Chlamydia muridarum	NC_002620	2005/12/04
Chlamydia trachomatis 434 Bu	NC_010287	2008/01/26
Chlamydia trachomatis A HAR-13	NC_007429	2007/01/23
Chlamydia trachomatis L2b UCH 1 proctitis	NC_010280	2008/01/26
Chlamydia trachomatis	NC_000117	2007/01/23
Chlamydophila abortus S26 3	NC_004552	2007/01/23
Chlamydophila caviae	NC_003361	2005/12/04
Chlamydophila felis Fe C-56	NC_007899	2006/03/16
Chlamydophila pneumoniae AR39	NC_002179	2005/12/04
Chlamydophila pneumoniae CWL029	NC_000922	2007/01/23
Chlamydophila pneumoniae J138	NC_002491	2005/12/04
Chlamydophila pneumoniae TW 183	NC_005043	2005/12/04
Chlorobaculum parvum NCIB 8327	NC_011027	2008/07/01
Chlorobium chlorochromatii CaD3	NC_007514	2007/04/25
Chlorobium limicola DSM 245	NC_010803	2008/09/11
Chlorobium phaeobacteroides BS1	NC_010831	2008/06/10
Chlorobium phaeobacteroides DSM 266	NC_008639	2007/07/31
Chlorobium tepidum TLS	NC_002932	2005/12/04
Chloroflexus aurantiacus J 10 fl	NC_010175	2008/03/18
Chloroherpeton thalassium ATCC 35110	NC_011026	2008/07/01
Chromobacterium violaceum	NC_005085	2005/12/04
Chromohalobacter salexigens DSM 3043	NC_007963	2007/01/23
Citrobacter koseri ATCC BAA-895	NC_009792	2007/09/14
Clavibacter michiganensis NCPPB 382	NC_009480	2007/05/23
Clavibacter michiganensis sepedonicus	NC_010407	2008/03/26
Clostridium acetobutylicum	NC_003030	2007/01/23
Clostridium beijerinckii NCIMB 8052	NC_009617	2007/06/29
Clostridium botulinum A3 Loch Maree	NC_010520	2008/03/24
Clostridium botulinum A ATCC 19397	NC_009697	2007/07/27
Clostridium botulinum A Hall	NC_009698	2007/07/27
Clostridium botulinum A	NC_009495	2008/01/07
Clostridium botulinum B1 Okra	NC_010516	2008/03/24
Clostridium botulinum B Eklund 17B	NC_010674	2008/05/10
Clostridium botulinum E3 Alaska E43	NC_010723	2008/09/11
Clostridium botulinum F Langeland	NC_009699	2007/07/27
Clostridium difficile 630	NC_009089	2008/01/07
Clostridium kluyveri DSM 555	NC_009706	2007/07/27
Clostridium novyi NT	NC_008593	2007/01/24
Clostridium perfringens ATCC 13124	NC_008261	2007/01/23
Clostridium perfringens	NC_003366	2005/12/04

Clostridium perfringens SM101	NC_008262, NC_008265	2007/04/25
Clostridium phytofermentans ISDg	NC_010001	2008/03/18
Clostridium tetani E88	NC_004557	2005/12/04
Clostridium thermocellum ATCC 27405	NC_009012	2007/02/17
Colwellia psychrerythraea 34H	NC_003910	2007/01/23
Coprothermobacter proteolyticus DSM 5265	NC_011295	2008/09/27
Corynebacterium diphtheriae	NC_002935	2007/01/23
Corynebacterium efficiens YS-314	NC_004369	2005/12/04
Corynebacterium glutamicum ATCC 13032 Bielefeld	NC_006958	2007/04/30
Corynebacterium glutamicum ATCC 13032 Kitasato	NC_003450	2007/01/23
Corynebacterium glutamicum R	NC_009342	2007/04/18
Corynebacterium jeikeium K411	NC_007164	2007/01/23
Corynebacterium urealyticum DSM 7109	NC_010545	2008/04/04
Coxiella burnetii CbuG Q212	NC_011527	2008/11/07
Coxiella burnetii CbuK Q154	NC_011528	2008/11/07
Coxiella burnetii Dugway 7E9-12	NC_009727	2007/12/14
Coxiella burnetii	NC_002971	2007/01/23
Coxiella burnetii RSA 331	NC_010117	2008/03/18
Cupriavidus taiwanensis	NC_010528, NC_010530	2008/07/17
Cyanobacteria bacterium Yellowstone A-Prime	NC_007775	2006/03/23
Cyanobacteria bacterium Yellowstone B-Prime	NC_007776	2006/03/23
Cytophaga hutchinsonii ATCC 33406	NC_008255	2006/08/30
Dechloromonas aromatica RCB	NC_007298	2007/01/23
Dehalococcoides BAV1	NC_009455	2007/05/18
Dehalococcoides CBDB1	NC_007356	2005/12/04
Dehalococcoides ethenogenes 195	NC_002936	2007/01/23
Deinococcus geothermalis DSM 11300	NC_008025	2006/05/09
Delftia acidovorans SPH-1	NC_010002	2008/03/18
Desulfitobacterium hafniense Y51	NC_007907	2006/03/16
Desulfotalea psychrophila LSv54	NC_006138	2005/12/04
Desulfotomaculum reducens MI-1	NC_009253	2007/03/30
Desulfovibrio desulfuricans G20	NC_007519	2007/01/23
Desulfovibrio vulgaris DP4	NC_008751	2007/01/05
Desulfovibrio vulgaris Hildenborough	NC_002937	2007/01/23
Dichelobacter nodosus VCS1703A	NC_009446	2007/05/09
Dictyoglomus thermophilum H 6 12	NC_011297	2008/09/27
Dinoroseobacter shibae DFL 12	NC_009952	2007/11/08
Ehrlichia canis Jake	NC_007354	2005/12/04
Ehrlichia chaffeensis Arkansas	NC_007799	2007/01/23
Ehrlichia ruminantium Gardel	NC_006831	2005/12/04
Ehrlichia ruminantium str. Welgevonden CIRAD	NC_006832	2005/12/04
Ehrlichia ruminantium Welgevonden UPSA	NC_005295	2007/01/23
Elusimicrobium minutum Pei191	NC_010644	2008/07/30
Enterobacter 638	NC_009436	2007/05/08
Enterobacter sakazakii ATCC BAA-894	NC_009778	2007/09/07
Enterococcus faecalis V583	NC_004668	2007/01/23
Erwinia carotovora atroseptica SCRI1043	NC_004547	2007/01/23
Erwinia tasmaniensis	NC_010694	2008/11/20
Erythrobacter litoralis HTCC2594	NC_007722	2006/01/20
Escherichia coli 536	NC_008253	2006/07/24
Escherichia coli APEC O1	NC_008563	2007/01/24
Escherichia coli C ATCC 8739	NC_010468	2008/05/09
Escherichia coli CFT073	NC_004431	2007/01/23
Escherichia coli E24377A	NC_009801	2007/09/14
Escherichia coli HS	NC_009800	2007/09/14
Escherichia coli K 12 substr DH10B	NC_010473	2008/04/22
Escherichia coli K12 substr MG1655	NC_000913	2008/05/19

Escherichia coli O157 H7 EC4115	NC_011353	2008/10/11
Escherichia coli O157H7 EDL933	NC_002655	2007/01/23
Escherichia coli O157H7	NC_002695	2007/01/23
Escherichia coli SE11	NC_011415	2008/10/24
Escherichia coli SMS 3 5	NC_010498	2008/07/30
Escherichia coli UTI89	NC_007946	2007/01/23
Escherichia coli W3110	AC_000091	2006/03/02
Exiguobacterium sibiricum 255 15	NC_010556	2008/04/06
Fervidobacterium nodosum Rt17-B1	NC_009718	2007/07/31
Finegoldia magna ATCC 29328	NC_010376	2008/03/14
Flavobacterium johnsoniae UW101	NC_009441	2007/05/08
Flavobacterium psychrophilum JIP02 86	NC_009613	2008/01/07
Francisella philomiragia ATCC 25017	NC_010336	2008/02/13
Francisella tularensis FSC 198	NC_008245	2008/02/07
Francisella tularensis holarctica FTA	NC_009749	2008/04/17
Francisella tularensis holarctica	NC_007880	2008/01/07
Francisella tularensis holarctica OSU18	NC_008369	2007/01/23
Francisella tularensis mediasiatica FSC147	NC_010677	2008/05/10
Francisella tularensis novicida U112	NC_008601	2007/01/23
Francisella tularensis tularensis	NC_006570	2007/01/23
Francisella tularensis WY96-3418	NC_009257	2007/03/30
Frankia alni ACN14a	NC_008278	2008/01/07
Frankia CcI3	NC_007777	2007/01/23
Frankia EAN1pec	NC_009921	2007/10/17
Fusobacterium nucleatum	NC_003454	2005/12/04
Geobacillus kaustophilus HTA426	NC_006510	2005/12/04
Geobacillus thermodenitrificans NG80-2	NC_009328	2007/04/03
Geobacter bemidjiensis Bem	NC_011146	2008/08/23
Geobacter lovleyi SZ	NC_010814	2008/06/07
Geobacter metallireducens GS-15	NC_007517	2007/01/23
Geobacter sulfurreducens	NC_002939	2007/01/23
Geobacter uraniumreducens Rf4	NC_009483	2008/05/08
Gloeobacter violaceus	NC_005125	2007/01/23
Gluconacetobacter diazotrophicus PAl 5	NC_010125	2007/12/10
Gluconobacter oxydans 621H	NC_006677	2005/12/04
Gramella forsetii KT0803	NC_008571	2006/12/30
Granulobacter bethesdensis CGDNIH1	NC_008343	2007/01/24
Haemophilus ducreyi 35000HP	NC_002940	2005/12/04
Haemophilus influenzae 86 028NP	NC_007146	2007/12/26
Haemophilus influenzae	NC_000907	2008/02/19
Haemophilus influenzae PittEE	NC_009566	2007/06/14
Haemophilus influenzae PittGG	NC_009567	2007/06/14
Haemophilus somnus 129PT	NC_008309	2007/01/24
Haemophilus somnus 2336	NC_010519	2008/03/25
Hahella chejuensis KCTC 2396	NC_007645	2005/12/15
Halobacterium salinarum R1	NC_010364	2008/03/14
Halobacterium sp	NC_002607	2005/12/04
Haloquadratum walsbyi	NC_008212	2006/08/25
Halorhodospira halophila SL1	NC_008789	2007/01/13
Helicobacter acinonychis Sheeba	NC_008229	2006/07/03
Helicobacter hepaticus	NC_004917	2005/12/04
Helicobacter pylori 26695	NC_000915	2007/01/23
Helicobacter pylori G27	NC_011333	2008/10/04
Helicobacter pylori HPAG1	NC_008086	2006/06/07
Helicobacter pylori J99	NC_000921	2007/01/23
Helicobacter pylori P12	NC_011498	2008/10/31
Helicobacter pylori Shi470	NC_010698	2008/10/12

Heliobacterium modesticaldum Ice1	NC_010337	2008/02/13
Herminiimonas arsenicoxydans	NC_009138	2008/01/07
Herpetosiphon aurantiacus ATCC 23779	NC_009972	2008/03/18
Hydrogenobaculum Y04AAS1	NC_011126	2008/08/07
Hyperthermus butylicus	NC_008818	2007/01/25
Hyphomonas neptunium ATCC 15444	NC_008358	2007/01/23
Idiomarina loihiensis L2TR	NC_006512	2005/12/04
Ignicoccus hospitalis KIN4 I	NC_009776	2007/09/07
Jannaschia CCS1	NC_007802	2007/01/23
Janthinobacterium Marseille	NC_009659	2007/07/25
Kineococcus radiotolerans SRS30216	NC_009664	2007/07/25
Klebsiella pneumoniae 342	NC_011283	2008/09/24
Klebsiella pneumoniae MGH 78578	NC_009648	2007/07/25
Kocuria rhizophila DC2201	NC_010617	2008/07/29
Lactobacillus acidophilus NCFM	NC_006814	2007/11/08
Lactobacillus brevis ATCC 367	NC_008497	2006/10/23
Lactobacillus casei ATCC 334	NC_008526	2006/10/23
Lactobacillus casei	NC_010999	2008/06/25
Lactobacillus delbrueckii bulgaricus ATCC BAA-365	NC_008529	2006/10/24
Lactobacillus delbrueckii bulgaricus	NC_008054	2006/06/14
Lactobacillus fermentum IFO 3956	NC_010610	2008/05/08
Lactobacillus gasseri ATCC 33323	NC_008530	2006/10/24
Lactobacillus helveticus DPC 4571	NC_010080	2007/12/04
Lactobacillus johnsonii NCC 533	NC_005362	2005/12/04
Lactobacillus plantarum	NC_004567	2005/12/04
Lactobacillus reuteri F275 JGI	NC_009513	2007/06/04
Lactobacillus reuteri F275 Kitasato	NC_010609	2008/11/15
Lactobacillus sakei 23K	NC_007576	2007/01/23
Lactobacillus salivarius UCC118	NC_007929	2008/01/07
Lactococcus lactis cremoris MG1363	NC_009004	2007/02/14
Lactococcus lactis cremoris SK11	NC_008527	2006/10/24
Lactococcus lactis	NC_002662	2007/01/23
Lawsonia intracellularis PHE MN1-00	NC_008011	2006/05/09
Legionella pneumophila Corby	NC_009494	2007/05/29
Legionella pneumophila Lens	NC_006369	2007/01/23
Legionella pneumophila Paris	NC_006368	2007/01/23
Legionella pneumophila Philadelphia 1	NC_002942	2007/01/24
Leifsonia xyli xyli CTCB0	NC_006087	2005/12/04
Leptothrix cholodnii SP 6	NC_010524	2008/03/28
Leuconostoc citreum KM20	NC_010471	2008/03/18
Leuconostoc mesenteroides ATCC 8293	NC_008531	2006/10/24
Listeria innocua	NC_003212	2007/01/23
Listeria monocytogenes 4b F2365	NC_002973	2007/01/23
Listeria monocytogenes	NC_003210	2007/01/23
Listeria welshimeri serovar 6b SLCC5334	NC_008555	2008/01/07
Lysinibacillus sphaericus C3 41	NC_010382	2008/03/13
Magnetococcus MC-1	NC_008576	2007/01/23
Magnetospirillum magneticum AMB-1	NC_007626	2005/12/07
Mannheimia succiniciproducens MBEL55E	NC_006300	2005/12/04
Maricaulis maris MCS10	NC_008347	2007/01/23
Marinobacter aquaeolei VT8	NC_008740	2007/01/10
Marinomonas MWYL1	NC_009654	2007/07/25
Mesoplasma florum L1	NC_006055	2005/12/04
Mesorhizobium BNC1	NC_008254	2007/01/23
Mesorhizobium loti	NC_002678	2005/12/04
Metallosphaera sedula DSM 5348	NC_009440	2007/05/08
Methanobacterium thermoautotrophicum	NC_000916	2007/05/09

Methanobrevibacter smithii ATCC 35061	NC_009515	2007/06/06
Methanococcoides burtonii DSM 6242	NC_007955	2007/01/23
Methanococcus aeolicus Nankai-3	NC_009635	2007/07/03
Methanococcus jannaschii	NC_000909, NC_001732, NC_001733	2008/02/19
Methanococcus maripaludis C5	NC_009135	2007/03/26
Methanococcus maripaludis C6	NC_009975	2008/03/18
Methanococcus maripaludis C7	NC_009637	2007/07/03
Methanococcus maripaludis S2	NC_005791	2007/01/23
Methanococcus vannielii SB	NC_009634	2007/07/03
Methanocorpusculum labreanum Z	NC_008942	2007/02/07
Methanoculleus marisnigri JR1	NC_009051	2007/02/26
Methanopyrus kandleri	NC_003551	2007/01/23
Methanosaeta thermophila PT	NC_008553	2007/01/23
Methanosarcina acetivorans	NC_003552	2005/12/04
Methanosarcina mazei	NC_003901	2007/01/23
Methanosphaera stadtmanae	NC_007681	2007/01/23
Methanospirillum hungatei JF-1	NC_007796	2007/01/23
Methylacidiphilum infernorum V4	NC_010794	2008/07/10
Methylibium petroleiphilum PM1	NC_008825	2007/01/30
Methylobacillus flagellatus KT	NC_007947	2006/04/11
Methylobacterium 4 46	NC_010511	2008/03/26
Methylobacterium extorquens PA1	NC_010172	2008/03/18
Methylobacterium populi BJ001	NC_010725	2008/09/11
Methylobacterium radiotolerans JCM 2831	NC_010505	2008/03/24
Methylococcus capsulatus Bath	NC_002977	2007/01/23
Microcystis aeruginosa NIES 843	NC_010296	2008/03/18
Moorella thermoacetica ATCC 39073	NC_007644	2007/01/23
Mycobacterium avium 104	NC_008595	2006/11/30
Mycobacterium avium paratuberculosis	NC_002944	2005/12/04
Mycobacterium bovis BCG Pasteur 1173P2	NC_008769	2007/01/11
Mycobacterium bovis	NC_002945	2007/01/24
Mycobacterium gilvum PYR-GCK	NC_009338	2007/04/16
Mycobacterium JLS	NC_009077	2007/03/01
Mycobacterium KMS	NC_008705	2006/12/23
Mycobacterium leprae	NC_002677	2007/01/23
Mycobacterium marinum M	NC_010612	2008/04/22
Mycobacterium MCS	NC_008146	2007/01/23
Mycobacterium smegmatis MC2 155	NC_008596	2006/11/30
Mycobacterium tuberculosis CDC1551	NC_002755	2005/12/04
Mycobacterium tuberculosis F11	NC_009565	2007/06/15
Mycobacterium tuberculosis H37Ra	NC_009525	2007/06/06
Mycobacterium tuberculosis H37Rv	NC_000962	2007/01/23
Mycobacterium ulcerans Agy99	NC_008611	2007/01/23
Mycobacterium vanbaalenii PYR-1	NC_008726	2006/12/29
Mycoplasma agalactiae PG2	NC_009497	2008/03/18
Mycoplasma arthritidis 158L3 1	NC_011025	2008/06/28
Mycoplasma capricolum ATCC 27343	NC_007633	2007/01/23
Mycoplasma gallisepticum	NC_004829	2005/12/04
Mycoplasma genitalium	NC_000908	2008/02/19
Mycoplasma hyopneumoniae 232	NC_006360	2005/12/04
Mycoplasma hyopneumoniae 7448	NC_007332	2005/12/04
Mycoplasma hyopneumoniae J	NC_007295	2005/12/04
Mycoplasma mobile 163K	NC_006908	2005/12/04
Mycoplasma mycoides	NC_005364	2007/03/09
Mycoplasma penetrans	NC_004432	2005/12/04
Mycoplasma pneumoniae	NC_000912	2005/12/04

Mycoplasma pulmonis	NC_002771	2005/12/04
Mycoplasma synoviae 53	NC_007294	2005/12/04
Myxococcus xanthus DK 1622	NC_008095	2007/01/23
Nanoarchaeum equitans	NC_005213	2007/01/23
Natranaerobius thermophilus JW NM WN LF	NC_010718	2008/09/11
Natronomonas pharaonis	NC_007426	2007/01/23
Neisseria gonorrhoeae FA 1090	NC_002946	2005/12/04
Neisseria gonorrhoeae NCCP11945	NC_011035	2008/07/12
Neisseria meningitidis 053442	NC_010120	2007/12/13
Neisseria meningitidis FAM18	NC_008767	2007/01/11
Neisseria meningitidis MC58	NC_003112	2005/12/04
Neisseria meningitidis Z2491	NC_003116	2005/12/04
Neorickettsia sennetsu Miyayama	NC_007798	2007/01/23
Nitratiruptor SB155-2	NC_009662	2007/07/26
Nitrobacter hamburgensis X14	NC_007964	2007/01/23
Nitrobacter winogradskyi Nb-255	NC_007406	2007/01/23
Nitrosococcus oceani ATCC 19707	NC_007484	2007/01/23
Nitrosomonas europaea	NC_004757	2007/01/23
Nitrosomonas eutropha C71	NC_008344	2007/01/23
Nitrosopumilus maritimus SCM1	NC_010085	2008/03/18
Nocardia farcinica IFM10152	NC_006361	2005/12/04
Nocardioides JS614	NC_008699	2006/12/22
Nostoc punctiforme PCC 73102	NC_010628	2008/06/11
Nostoc sp	NC_003272	2008/03/18
Novosphingobium aromaticivorans DSM 12444	NC_007794	2007/01/23
Oceanobacillus iheyensis	NC_004193	2008/03/18
Oenococcus oeni PSU-1	NC_008528	2006/10/23
Oligotropha carboxidovorans OM5	NC_011386	2008/10/23
Onion yellows phytoplasma	NC_005303	2005/12/04
Opitutus terrae PB90 1	NC_010571	2008/04/12
Orientia tsutsugamushi Boryong	NC_009488	2007/05/24
Orientia tsutsugamushi Ikeda	NC_010793	2008/06/02
Parabacteroides distasonis ATCC 8503	NC_009615	2007/06/29
Parachlamydia sp UWE25	NC_005861	2005/12/19
Parvibaculum lavamentivorans DS-1	NC_009719	2007/07/31
Pasteurella multocida	NC_002663	2005/12/04
Pediococcus pentosaceus ATCC 25745	NC_008525	2006/10/23
Pelobacter carbinolicus	NC_007498	2006/04/01
Pelobacter propionicus DSM 2379	NC_008609	2007/01/23
Pelodictyon luteolum DSM 273	NC_007512	2007/01/23
Pelodictyon phaeoclathratiforme BU 1	NC_011060	2008/07/21
Pelotomaculum thermopropionicum SI	NC_009454	2007/05/18
Petrotoga mobilis SJ95	NC_010003	2008/03/18
Phenylobacterium zucineum HLK1	NC_011144	2008/08/22
Photorhabdus luminescens	NC_005126	2007/01/23
Picrophilus torridus DSM 9790	NC_005877	2007/01/24
Pirellula sp	NC_005027	2007/01/23
Polaromonas JS666	NC_007948	2007/01/23
Polaromonas naphthalenivorans CJ2	NC_008781	2007/01/11
Polynucleobacter necessarius STIR1	NC_010531	2008/04/01
Polynucleobacter QLW-P1DMWA-1	NC_009379	2007/04/25
Porphyromonas gingivalis ATCC 33277	NC_010729	2008/06/12
Porphyromonas gingivalis W83	NC_002950	2005/12/04
Prochlorococcus marinus AS9601	NC_008816	2007/01/23
Prochlorococcus marinus CCMP1375	NC_005042	2005/12/04
Prochlorococcus marinus MED4	NC_005072	2007/01/23
Prochlorococcus marinus MIT 9211	NC_009976	2008/03/18

Prochlorococcus marinus MIT 9215	NC_009840	2007/09/22
Prochlorococcus marinus MIT 9301	NC_009091	2007/03/07
Prochlorococcus marinus MIT 9303	NC_008820	2007/01/24
Prochlorococcus marinus MIT 9312	NC_007577	2007/01/23
Prochlorococcus marinus MIT9313	NC_005071	2007/01/23
Prochlorococcus marinus MIT 9515	NC_008817	2007/01/23
Prochlorococcus marinus NATL1A	NC_008819	2007/01/24
Prochlorococcus marinus NATL2A	NC_007335	2007/12/26
Propionibacterium acnes KPA171202	NC_006085	2005/12/04
Prosthecochloris aestuarii DSM 271	NC_011059	2008/07/21
Prosthecochloris vibrioformis DSM 265	NC_009337	2007/04/16
Proteus mirabilis	NC_010554	2008/08/27
Pseudoalteromonas atlantica T6c	NC_008228	2007/01/23
Pseudoalteromonas haloplanktis TAC125	NC_007481	2008/10/01
Pseudomonas aeruginosa	NC_002516	2006/07/24
Pseudomonas aeruginosa PA7	NC_009656	2007/07/25
Pseudomonas aeruginosa UCBPP-PA14	NC_008463	2007/01/24
Pseudomonas entomophila L48	NC_008027	2008/01/07
Pseudomonas fluorescens Pf0 1	NC_007492	2008/09/19
Pseudomonas fluorescens Pf-5	NC_004129	2007/01/23
Pseudomonas mendocina ymp	NC_009439	2007/05/08
Pseudomonas putida F1	NC_009512	2007/06/04
Pseudomonas putida GB 1	NC_010322	2008/03/18
Pseudomonas putida KT2440	NC_002947	2007/01/23
Pseudomonas putida W619	NC_010501	2008/03/25
Pseudomonas stutzeri A1501	NC_009434	2007/05/08
Pseudomonas syringae phaseolicola 1448A	NC_005773	2005/12/04
Pseudomonas syringae pv B728a	NC_007005	2005/12/04
Pseudomonas syringae tomato DC3000	NC_004578	2005/12/04
Psychrobacter arcticum 273-4	NC_007204	2005/12/04
Psychrobacter cryohalolentis K5	NC_007969	2007/01/23
Psychrobacter PRwf-1	NC_009524	2007/06/06
Psychromonas ingrahamii 37	NC_008709	2006/12/27
Pyrobaculum aerophilum	NC_003364	2007/01/23
Pyrobaculum arsenaticum DSM 13514	NC_009376	2007/04/25
Pyrobaculum calidifontis JCM 11548	NC_009073	2007/03/01
Pyrobaculum islandicum DSM 4184	NC_008701	2006/12/23
Pyrococcus abyssi	NC_000868	2005/12/04
Pyrococcus furiosus	NC_003413	2007/01/23
Pyrococcus horikoshii	NC_000961	2007/01/23
Ralstonia solanacearum	NC_003295	2007/01/23
Renibacterium salmoninarum ATCC 33209	NC_010168	2007/12/26
Rhizobium etli CFN 42	NC_007761	2007/01/23
Rhizobium etli CIAT 652	NC_010994	2008/06/21
Rhizobium leguminosarum bv trifolii WSM2304	NC_011369	2008/10/17
Rhizobium leguminosarum bv viciae 3841	NC_008380	2008/01/07
Rhodobacter sphaeroides ATCC 17025	NC_009428	2007/05/08
Rhodococcus RHA1	NC_008268	2006/07/31
Rhodoferax ferrireducens T118	NC_007908	2007/01/23
Rhodopseudomonas palustris BisA53	NC_008435	2007/01/23
Rhodopseudomonas palustris BisB18	NC_007925	2007/01/23
Rhodopseudomonas palustris BisB5	NC_007958	2007/01/23
Rhodopseudomonas palustris CGA009	NC_005296	2007/01/23
Rhodopseudomonas palustris HaA2	NC_007778	2007/01/24
Rhodopseudomonas palustris TIE 1	NC_011004	2008/06/24
Rhodospirillum centenum SW	NC_011420	2008/10/25
Rhodospirillum rubrum ATCC 11170	NC_007643	2007/01/24

Rickettsia akari Hartford	NC_009881	2007/10/04
Rickettsia bellii OSU 85-389	NC_009883	2007/10/04
Rickettsia bellii RML369-C	NC_007940	2007/01/24
Rickettsia canadensis McKiel	NC_009879	2007/10/03
Rickettsia conorii	NC_003103	2007/01/24
Rickettsia felis URRWXCal2	NC_007109	2007/01/24
Rickettsia massiliae MTU5	NC_009900	2007/10/10
Rickettsia prowazekii	NC_000963	2007/01/24
Rickettsia rickettsii Iowa	NC_010263	2008/01/19
Rickettsia rickettsii Sheila Smith	NC_009882	2007/10/04
Rickettsia typhi wilmington	NC_006142	2007/01/24
Roseiflexus castenholzii DSM 13941	NC_009767	2007/09/05
Roseiflexus RS-1	NC_009523	2007/06/06
Roseobacter denitrificans OCh 114	NC_008209	2006/07/25
Rubrobacter xylanophilus DSM 9941	NC_008148	2007/01/24
Saccharophagus degradans 2-40	NC_007912	2007/01/24
Saccharopolyspora erythraea NRRL 2338	NC_009142	2007/03/26
Salinibacter ruber DSM 13855	NC_007677	2007/01/24
Salinispora arenicola CNS-205	NC_009953	2007/11/08
Salinispora tropica CNB-440	NC_009380	2007/04/25
Salmonella enterica arizonae serovar 62 z4 z23	NC_010067	2008/03/18
Salmonella enterica Choleraesuis	NC_006905	2007/01/24
Salmonella enterica Paratypi ATCC 9150	NC_006511	2007/01/24
Salmonella enterica serovar Agona SL483	NC_011149	2008/08/26
Salmonella enterica serovar Dublin CT 02021853	NC_011205	2008/09/06
Salmonella enterica serovar Enteritidis P125109	NC_011294	2008/10/02
Salmonella enterica serovar Gallinarum 287 91	NC_011274	2008/09/20
Salmonella enterica serovar Heidelberg SL476	NC_011083	2008/07/25
Salmonella enterica serovar Newport SL254	NC_011080	2008/07/25
Salmonella enterica serovar Paratyphi A AKU 12601	NC_011147	2008/09/04
Salmonella enterica serovar Paratyphi B SPB7	NC_010102	2008/03/18
Salmonella enterica serovar Schwarzengrund CVM19633	NC_011094	2008/07/31
Salmonella enterica serovar Typhi Ty2	NC_004631	2007/01/23
Salmonella typhimurium LT2	NC_003197	2005/12/04
Salmonella typhi	NC_003198	2005/12/04
Serratia proteamaculans 568	NC_009832	2007/09/20
Shewanella amazonensis SB2B	NC_008700	2006/12/22
Shewanella baltica OS155	NC_009052	2007/02/26
Shewanella baltica OS185	NC_009665	2007/07/25
Shewanella baltica OS195	NC_009997	2008/03/18
Shewanella denitrificans OS217	NC_007954	2007/01/24
Shewanella frigidimarina NCIMB 400	NC_008345	2007/01/24
Shewanella halifaxensis HAW EB4	NC_010334	2008/03/18
Shewanella loihica PV-4	NC_009092	2007/03/09
Shewanella MR-4	NC_008321	2007/01/24
Shewanella MR-7	NC_008322	2007/01/24
Shewanella oneidensis	NC_004347	2005/12/04
Shewanella pealeana ATCC 700345	NC_009901	2007/10/10
Shewanella piezotolerans WP3	NC_011566	2008/11/14
Shewanella putrefaciens CN-32	NC_009438	2007/05/08
Shewanella sediminis HAW-EB3	NC_009831	2007/09/20
Shewanella W3-18-1	NC_008750	2007/01/05
Shewanella woodyi ATCC 51908	NC_010506	2008/03/25
Shigella boydii CDC 3083 94	NC_010658	2008/05/09
Shigella boydii Sb227	NC_007613	2005/12/04
Shigella dysenteriae	NC_007606	2005/12/07
Shigella flexneri 2a 2457T	NC_004741	2007/01/24

Shigella flexneri 2a	NC_004337	2007/01/24
Shigella flexneri 5 8401	NC_008258	2006/07/28
Shigella sonnei Ss046	NC_007384	2005/12/04
Silicibacter pomeroyi DSS-3	NC_003911	2005/12/04
Silicibacter TM1040	NC_008044	2007/01/24
Sinorhizobium medicae WSM419	NC_009636	2007/07/03
Sinorhizobium meliloti	NC_003047	2007/01/24
Sodalis glossinidius morsitans	NC_007712	2006/01/18
Solibacter usitatus Ellin6076	NC_008536	2007/01/24
Sorangium cellulosum So ce 56	NC_010162	2007/12/14
Sphingomonas wittichii RW1	NC_009511	2007/06/04
Sphingopyxis alaskensis RB2256	NC_008048	2007/01/24
Staphylococcus aureus aureus MRSA252	NC_002952	2007/01/24
Staphylococcus aureus aureus MSSA476	NC_002953	2007/01/24
Staphylococcus aureus COL	NC_002951	2007/01/24
Staphylococcus aureus JH1	NC_009632	2007/07/03
Staphylococcus aureus JH9	NC_009487	2007/05/23
Staphylococcus aureus Mu3	NC_009782	2007/09/07
Staphylococcus aureus Mu50	NC_002758	2007/01/24
Staphylococcus aureus MW2	NC_003923	2005/12/04
Staphylococcus aureus N315	NC_002745	2007/01/24
Staphylococcus aureus NCTC 8325	NC_007795	2006/02/18
Staphylococcus aureus Newman	NC_009641	2007/07/07
Staphylococcus aureus RF122	NC_007622	2007/01/24
Staphylococcus aureus USA300	NC_007793	2007/01/24
Staphylococcus aureus USA300 TCH1516	NC_010079	2008/03/18
Staphylococcus epidermidis ATCC 12228	NC_004461	2005/12/04
Staphylococcus epidermidis RP62A	NC_002976	2007/01/24
Staphylococcus haemolyticus	NC_007168	2005/12/04
Staphylococcus saprophyticus	NC_007350	2006/03/02
Stenotrophomonas maltophilia K279a	NC_010943	2008/06/18
Stenotrophomonas maltophilia R551 3	NC_011071	2008/07/23
Streptococcus agalactiae 2603	NC_004116	2007/01/24
Streptococcus agalactiae A909	NC_007432	2007/01/24
Streptococcus agalactiae NEM316	NC_004368	2007/01/24
Streptococcus equi zooepidemicus MGCS10565	NC_011134	2008/10/01
Streptococcus gordonii Challis substr CH1	NC_009785	2007/09/14
Streptococcus mutans	NC_004350	2005/12/04
Streptococcus pneumoniae CGSP14	NC_010582	2008/04/12
Streptococcus pneumoniae D39	NC_008533	2006/10/24
Streptococcus pneumoniae G54	NC_011072	2008/07/24
Streptococcus pneumoniae Hungary19A 6	NC_010380	2008/03/18
Streptococcus pneumoniae R6	NC_003098	2005/12/04
Streptococcus pneumoniae TIGR4	NC_003028	2008/07/12
Streptococcus pyogenes M1 GAS	NC_002737	2007/01/24
Streptococcus pyogenes Manfredo	NC_009332	2008/01/07
Streptococcus pyogenes MGAS10270	NC_008022	2006/05/09
Streptococcus pyogenes MGAS10394	NC_006086	2005/12/04
Streptococcus pyogenes MGAS10750	NC_008024	2006/05/09
Streptococcus pyogenes MGAS2096	NC_008023	2006/05/09
Streptococcus pyogenes MGAS315	NC_004070	2007/01/24
Streptococcus pyogenes MGAS5005	NC_007297	2007/01/24
Streptococcus pyogenes MGAS6180	NC_007296	2005/12/04
Streptococcus pyogenes MGAS8232	NC_003485	2007/01/24
Streptococcus pyogenes MGAS9429	NC_008021	2006/05/09
Streptococcus pyogenes NZ131	NC_011375	2008/10/17
Streptococcus pyogenes SSI-1	NC_004606	2007/01/24

Streptococcus sanguinis SK36	NC_009009	2007/02/16
Streptococcus suis 05ZYH33	NC_009442	2007/05/08
Streptococcus suis 98HAH33	NC_009443	2007/05/08
Streptococcus thermophilus CNRZ1066	NC_006449	2005/12/04
Streptococcus thermophilus LMD-9	NC_008532	2006/10/24
Streptococcus thermophilus LMG 18311	NC_006448	2005/12/04
Streptomyces avermitilis	NC_003155	2007/12/26
Streptomyces coelicolor	NC_003888	2007/01/24
Streptomyces griseus NBRC 13350	NC_010572	2008/04/12
Sulfurihydrogenibium YO3AOP1	NC_010730	2008/06/12
Sulfurovum NBC37-1	NC_009663	2007/07/26
Symbiobacterium thermophilum IAM14863	NC_006177	2005/12/04
Synechococcus CC9311	NC_008319	2007/01/24
Synechococcus CC9605	NC_007516	2007/01/24
Synechococcus CC9902	NC_007513	2005/12/04
Synechococcus elongatus PCC 6301	NC_006576	2005/12/04
Synechococcus elongatus PCC 7942	NC_007604	2007/01/24
Synechococcus PCC 7002	NC_010475	2008/03/18
Synechococcus RCC307	NC_009482	2007/05/23
Synechococcus sp WH8102	NC_005070	2007/01/24
Synechococcus WH 7803	NC_009481	2007/05/23
Synechocystis PCC6803	NC_000911	2007/01/24
Syntrophobacter fumaroxidans MPOB	NC_008554	2007/01/24
Syntrophomonas wolfei Goettingen	NC_008346	2007/01/24
Syntrophus aciditrophicus SB	NC_007759	2006/04/19
Thermoanaerobacter pseudethanolicus ATCC 33223	NC_010321	2008/03/18
Thermoanaerobacter tengcongensis	NC_003869	2007/01/24
Thermoanaerobacter X514	NC_010320	2008/03/18
Thermobifida fusca YX	NC_007333	2007/01/24
Thermococcus kodakaraensis KOD1	NC_006624	2005/12/04
Thermococcus onnurineus NA1	NC_011529	2008/11/07
Thermodesulfovibrio yellowstonii DSM 11347	NC_011296	2008/09/27
Thermofilum pendens Hrk 5	NC_008698	2006/12/22
Thermoplasma acidophilum	NC_002578	2007/01/24
Thermoplasma volcanium	NC_002689	2006/12/21
Thermosipho melanesiensis BI429	NC_009616	2007/06/29
Thermosynechococcus elongatus	NC_004113	2007/01/24
Thermotoga lettingae TMO	NC_009828	2007/11/20
Thermotoga maritima	NC_000853	2007/01/24
Thermotoga petrophila RKU-1	NC_009486	2007/05/23
Thermotoga RQ2	NC_010483	2008/06/02
Thermus thermophilus HB27	NC_005835	2005/12/04
Thermus thermophilus HB8	NC_006461	2007/01/24
Thiobacillus denitrificans ATCC 25259	NC_007404	2007/01/24
Thiomicrospira crunogena XCL-2	NC_007520	2007/01/24
Thiomicrospira denitrificans ATCC 33889	NC_007575	2008/01/26
Treponema denticola ATCC 35405	NC_002967	2005/12/04
Treponema pallidum	NC_000919	2007/01/24
Treponema pallidum SS14	NC_010741	2008/06/12
Trichodesmium erythraeum IMS101	NC_008312	2007/01/24
Tropheryma whipplei TW08 27	NC_004551	2007/01/24
Tropheryma whipplei Twist	NC_004572	2005/12/04
uncultured Termite group 1 bacterium phylotype Rs D17	NS_000191	2008/11/22
Ureaplasma parvum serovar 3 ATCC 27815	NC_010503	2008/03/26
Ureaplasma urealyticum	NC_002162	2005/12/04
Ureaplasma urealyticum serovar 10 ATCC 33699	NC_011374	2008/10/17
Verminephrobacter eiseniae EF01-2	NC_008786	2007/01/11

Vibrio harveyi ATCC BAA-1116	NC_009783	2008/09/26
Vibrio vulnificus CMCP6	NC_004459	2008/09/25
Wigglesworthia brevipalpis	NC_004344	2007/01/24
Wolbachia endosymbiont of Brugia malayi TRS	NC_006833	2005/12/04
Wolbachia endosymbiont of Culex quinquefasciatus Pel	NC_010981	2008/11/20
Wolbachia endosymbiont of Drosophila melanogaster	NC_002978	2007/01/24
Wolinella succinogenes	NC_005090	2005/12/04
Xanthobacter autotrophicus Py2	NC_009720	2007/07/31
Xanthomonas campestris 8004	NC_007086	2007/01/24
Xanthomonas campestris ATCC 33913	NC_003902	2007/01/24
Xanthomonas campestris B100	NC_010688	2008/09/08
Xanthomonas campestris vesicatoria 85-10	NC_007508	2007/01/24
Xanthomonas citri	NC_003919	2007/01/24
Xanthomonas oryzae KACC10331	NC_006834	2005/12/04
Xanthomonas oryzae MAFF 311018	NC_007705	2007/01/24
Xanthomonas oryzae PXO99A	NC_010717	2008/09/11
Xylella fastidiosa M12	NC_010513	2008/03/24
Xylella fastidiosa M23	NC_010577	2008/04/12
Xylella fastidiosa	NC_002488	2007/01/24
Xylella fastidiosa Temecula1	NC_004556	2005/12/03
Yersinia enterocolitica 8081	NC_008800	2008/01/07
Yersinia pestis Angola	NC_010159	2008/03/18
Yersinia pestis Antiqua	NC_008150	2007/01/24
Yersinia pestis biovar Microtus 91001	NC_005810	2008/09/11
Yersinia pestis CO92	NC_003143	2005/12/03
Yersinia pestis KIM	NC_004088	2005/12/03
Yersinia pestis Nepal516	NC_008149	2007/01/24
Yersinia pestis Pestoides F	NC_009381	2007/04/25
Yersinia pseudotuberculosis IP 31758	NC_009708	2007/07/27
Yersinia pseudotuberculosis IP32953	NC_006155	2007/01/24
Yersinia pseudotuberculosis PB1	NC_010634	2008/07/31
Yersinia pseudotuberculosis YPIII	NC_010465	2008/03/18

Table 1: Data sources: The protein sets of these species were downloaded from `ftp://ftp.ncbi.nih.gov/genomes/Bacteria/` and used within the domain-wide common approach.
